# A randomized trial to evaluate the effects of a supervised exercise program on insomnia in patients with non-metastatic breast cancer undergoing chemotherapy: design of the FATSOMCAN study

**DOI:** 10.1186/s12885-023-10902-6

**Published:** 2023-05-17

**Authors:** Chloé Drozd, Elsa Curtit, Quentin Jacquinot, Charlène Marquine, Laura Mansi, Loïc Chaigneau, Erion Dobi, Julien Viot, Guillaume Meynard, Marie-Justine Paillard, Morgan Goujon, Pauline Roux, Dewi Vernerey, Valérie Gillet, Hubert Bourdin, Silvio Galli, Nathalie Meneveau, Fabienne Mougin

**Affiliations:** 1 Sports Science Faculty, University of Franche-Comté, Besançon, 25000 France; 2 Research Unit EA3920, University of Franche-Comté, Besançon, 25000 France; 3 Sleep Medicine Center, Ellipse, Besançon, 25000 France; 4 Department of Medical Oncology, University Hospital, Besançon, 25000 France; 5 INSERM U1098 RIGHT, University of Franche-Comté, Besançon, France; 6 Regional Federative Cancer Institute of Franche-Comté, Besançon, France; 7 Department of Physiology-Functional Explorations, University Hospital, Besançon, 25000 France; 8 Methodology and Quality of Life Unit, UMR 1098, University Hospital, Besançon, 25000 France; 9 Research Unit EA481, Unit of Sleep Disorder, University of Franche-Comté, Besançon, 25000 France; 10 Department of Neurology, University Hospital, Besançon, 25000 France

**Keywords:** Breast cancer, Insomnia, Sleep, Polysomnography, Fatigue, Exercise, Supportive care, Study protocol

## Abstract

**Background:**

Up to 70% of breast cancer patients report symptoms of insomnia during and after treatment. Despite the ubiquity of insomnia symptoms, they are under-screened, under-diagnosed and poorly managed in breast cancer patients. Sleep medications treat symptoms but are ineffective to cure insomnia. Other approaches such as cognitive behavioral therapy for insomnia, relaxation through yoga and mindfulness are often not available for patients and are complex to implement. An aerobic exercise program could be a promising treatment and a feasible option for insomnia management in breast cancer patients, but few studies have investigated the effects of such a program on insomnia.

**Methods:**

This multicenter, randomized clinical trial evaluate the effectiveness of a moderate to high intensity physical activity program (45 min, 3 times per week), lasting 12 weeks, in minimizing insomnia, sleep disturbances, anxiety/depression, fatigue, and pain, and in enhancing cardiorespiratory fitness. Patients with breast cancer be recruited from six hospitals in France and randomly allocated to either the “training” or the “control” group. Baseline assessments include questionnaires [Insomnia Severity Index (ISI), Pittsburgh Sleep Quality Index questionnaire (PSQI), Hospital Anxiety Depression Scale (HADS), Epworth Sleepiness Scale (ESS)], home polysomnography (PSG), and 7-day actigraphy coupled with completion of a sleep diary. Assessments are repeated at the end of training program and at six-month follow-up.

**Discussion:**

This clinical trial will provide additional evidence regarding the effectiveness of physical exercise in minimizing insomnia during and after chemotherapy. If shown to be effective, exercise intervention programs will be welcome addition to the standard program of care offered to patients with breast cancer receiving chemotherapy.

**Trial registration:**

: National Clinical Trials Number (NCT04867096).

## Background

Breast cancer is the most commonly diagnosed cancer in women, with an incidence of 11.6% worldwide in 2018 [1], although the mortality rate is declining in many developed countries [2]. In breast cancer patients, a common sleep disorder is insomnia [2]. A large, prospective study found that during chemotherapy for breast cancer, 43% of patients met the criteria for insomnia syndrome, and patients with breast cancer had the highest number of overall insomnia complaints [3]. About 40–70% of breast cancer survivors report sleep problems even years after diagnosis, depending on the diagnostic criteria applied [4, 5]. The Diagnostic and Statistical Manual of Mental Disorders, 5th Edition (DSM-5) defines insomnia as a dissatisfaction with sleep quantity or quality, associated with difficulty initiating or maintaining sleep, with the inability to return to sleep, present for at least 3 months, and occurring at least 3 nights per week despite adequate opportunity for sleep [6]. Insomnia can be caused or exacerbated by multiple treatment-related factors, including endocrine therapy and hot flashes, pain and discomfort from local therapy, and fear of recurrence. Insomnia leads to impaired psychological and physical health and may even increase mortality in breast cancer patients [7–9]. Despite its prevalence and impact, insomnia is rarely addressed in the oncology clinic. However, treatment approaches for insomnia are available to clinicians treating patients with breast cancer. There is now a considerable body of evidence supporting the use of psychosocial interventions and behavioral treatments, such as cognitive behavioral therapy for insomnia (CBT-I), yoga, as well as mind-body programs. Indeed, CBT-I is recommended as a first-line treatment for chronic insomnia [10]. Nevertheless, further treatment options for insomnia are necessary, since CBT-I is often unavailable for patients, and may be complex to implement in clinical routine, while poor adherence can decrease its effectiveness [11].

A physical exercise program might be a promising treatment option for preventing cancer-related insomnia, and appears to be safe. Previous studies showed that improved sleep quality positively impacts on quality of life in cancer patients [12] and that the total volume of weekly exercise might influence sleep [13].

Moreover, exercise is recommended during treatment with curative intent. Aerobic and resistance exercise has been shown to reduce fatigue, preserve cardiorespiratory fitness and physical functioning, as well as improve quality of life and depression symptoms [14]. Several trials demonstrated that exercise interventions are feasible in early breast cancer patients and have a low risk of severe adverse events [15, 16].

However, few studies have explored the efficacy of an exercise program for cancer-related insomnia among patients undergoing breast cancer treatments. In this regard, we hypothesize that exercise programs can have a beneficial effect on insomnia for breast cancer patients.

We designed the present randomized FATSOMCAN trial, to examine, in non-metastatic early breast cancer patients with an insomnia diagnosis, the effect on sleep quantity of an individualized, intermittent aerobic exercise training regimen (45 min, 3 times a week), comprising both moderate and high intensity exercise, for a period of 3 months. Secondary objectives are to evaluate the effects of this supervised exercise program on other parameters such as sleep disorders, sleepiness, anxiety/depression, core body temperature and salivary melatonin rhythm. Furthermore, fatigue, pain, inflammatory and cardiorespiratory fitness variables, and level of physical activity (PA) are assessed.

We hypothesize that a supervised exercise program will reduce insomnia and improve the quality and quantity of sleep. Exercise will increase cardio-respiratory fitness, with an improvement in inflammatory variables. We suppose that these modifications will be accompanied by a reduction in sleepiness, anxiety/depression, fatigue and pain. Moreover, we hypothesize that patients who participate in the supervised exercise program will maintain these benefits at the three-month follow-up.

## Methods

**Fig. 1:**
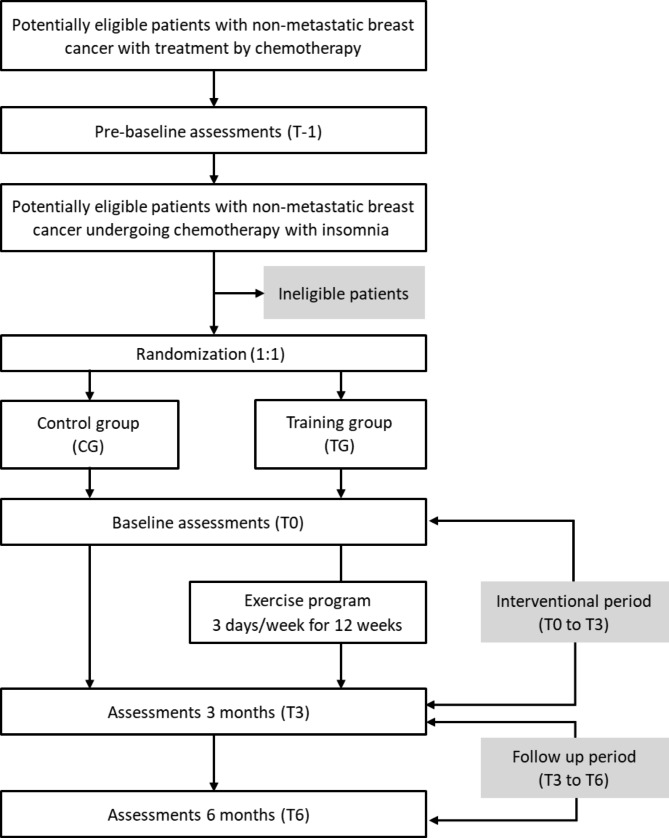
Flowchart for the FATSOMCAN trial

The FATSOMCAN study was approved by the Ethics Committee “Comité de Protection des Personnes Ouest III” on 15/09/2020 under the number 2020-A00511-38, and by the National Health Products Safety Agency (N° IDRCB 2020-A00511-38). The study is registered on http://www.clinicaltrials.gov under the identifier NCT04867096. Patient recruitment and data collection started in February 2021. The design of the trial is displayed in Fig. 1.

### Recruitment

Six medical oncology centers have been identified in Franche-Comté (France) for the recruitment. Women are recruited and deemed eligible if they meet the following criteria: (1) Aged over 18 and ≤ 65 years; (2) early breast cancer confirmed histologically; (3) undergoing first adjuvant or neoadjuvant sequential chemotherapy with anthracyclines and taxanes; (4) diagnosed as having insomnia; (5) no contraindication to performing physical activity (PA); (6) and either using appropriate contraception or menopaused.

Patients are not eligible in case of: (1) metastases; (2) treatment with melatonin or hypnotics; (3) no diagnosis of insomnia; (4) documented depression; (5) resting oxygen saturation (SaO_2_) ≤ 92%; (6) autoimmune disease (systemic lupus erythematosus, rheumatoid arthritis); (7) symptomatic osteoarthritis, cardiovascular disease (angina or uncontrolled hypertension) or lung disease (chronic obstructive pulmonary disease); (8) malnutrition (body mass index < 18 kg.m^− 2^) or weight loss of > 10% during the previous 3 months; (9) psychiatric or cognitive disorders; (10) pregnant or breastfeeding patients.

Potentially eligible patients are identified during a multidisciplinary tumor board (MTB) composed of oncologists, surgeons and radiotherapists. The choice of the type of surgery, chemotherapy and radiotherapy regimens is left at the discretion of the treating physician.

Potential candidates receive the study information when they come to the hospital for clinical follow-up. If they are interested in participating in the study, a researcher contact them to conduct a phone screen (see below).

Prior to participation, all participants must provide written informed consent.

The participants are instructed to maintain their regular exercise activities, but not to participate in any new exercise program during the study period other than the program proposed in the context of the study.

### Study outcomes

**Fig. 2:**
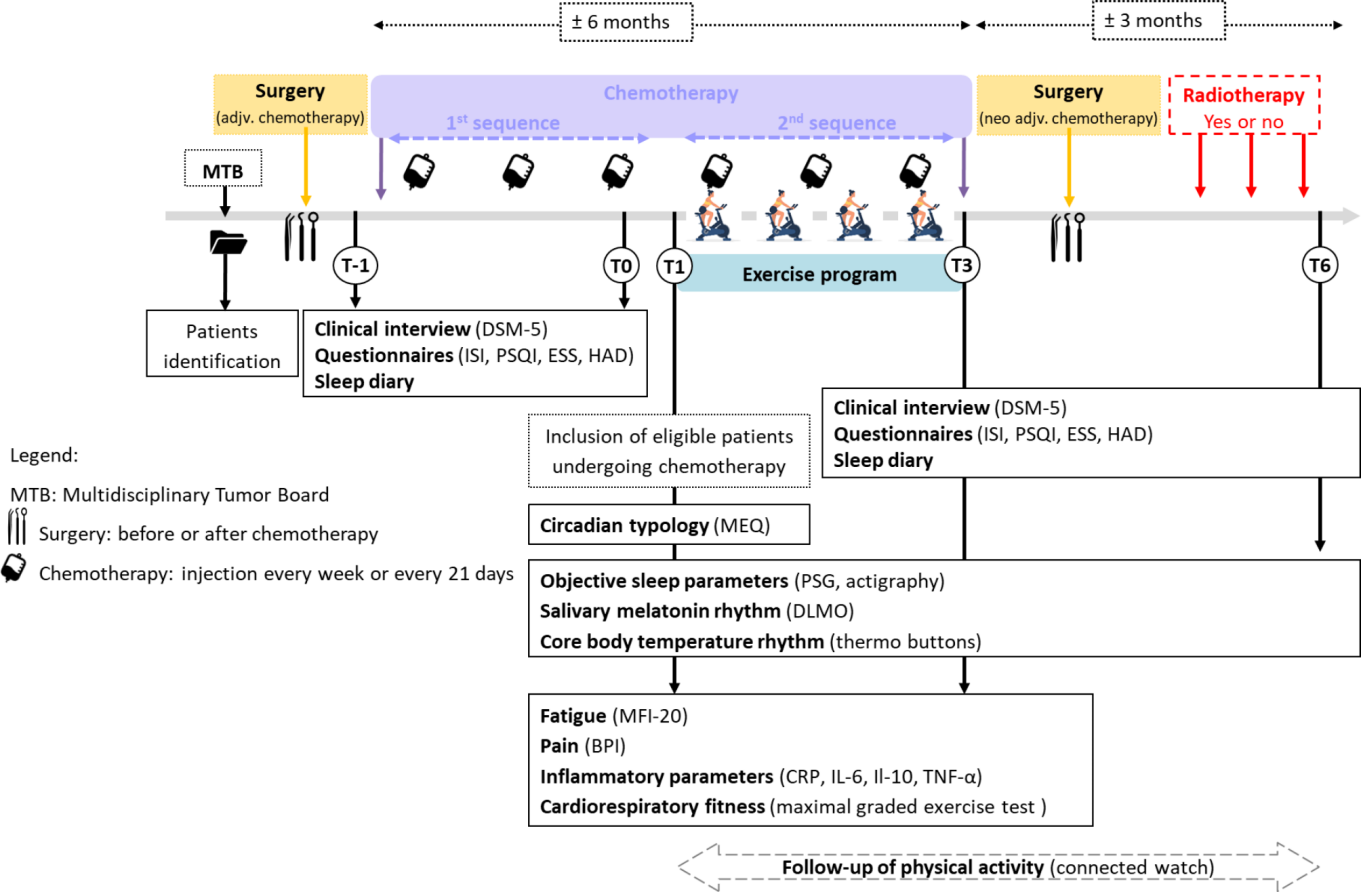
Representation of treatment schedule and different times of evaluation and questionnaire completion

The experimental protocol is described in Fig. 2.

At T-1, all potentially eligible patients have a clinical interview according to the DSM-5 to diagnose the presence of insomnia before treatment by chemotherapy. They also complete the study questionnaires [i.e., the Insomnia Severity Index (ISI), the Pittsburgh Sleep Quality Index (PSQI), the Epworth Sleepiness Scale (ESS) and Hospital Anxiety and Depression Scale (HADS)] and a sleep diary for 2 weeks. The interview, questionnaires and sleep diary constitute the baseline values before inclusion. A clinical examination, including medical history, use of analgesics (type, level, dose), lifestyle habits (work, family environment, physical activity, smoking, alcohol), is also carried out.

At T0, one week after completion of the first sequence of the chemotherapy, patients have a clinical interview, complete the questionnaires and sleep diary again. If insomnia is confirmed, patients can then be included.

At T1, at the end of the 3-month exercise intervention (T3) and at 6 months (T6) of the follow up period, objective sleep parameters, salivary melatonin and core body temperature rhythm are evaluated. At T1 and T3, fatigue, pain, inflammatory variables and cardiorespiratory fitness are assessed. At T3, DMS-5 interview, questionnaires and sleep diary are completed again. From T1 to T6, the level of PA is followed (Fig. 2).

### Randomization

At T1, patients are randomly assigned (in a 1:1 ratio) to one of the two groups, namely either to receive standard oncological care alone (Control Group: CG) or standard care plus an exercise program (Training Group: TG), which starts at the halfway point through the chemotherapy protocol. During the follow-up period (T3-T6), both groups follow standard oncological care without supervised PA. Furthermore, the level of PA, without guidelines, is assessed by a connected watch.

### Primary endpoint

The primary endpoint is a reduction in insomnia assessed by an increase in total sleep time (TST).

### Secondary endpoints

Secondary endpoints are a decrease of insomnia severity, an improvement in sleep quality, a decrease in sleepiness, anxiety/depression, pain, fatigue, inflammatory variables, and an enhancement of cardiorespiratory fitness.

#### Sleep architecture and composition

Sleep architecture and composition are assessed by an ambulatory polysomnography, which represents the gold standard to objectively measure TST, sleep patterns and diagnose sleep disorders (e.g., obstructive sleep apnea, periodic limb movements). Patients undergo one night of home-based polysomnography (PSG) (Morpheus, Micromed, Italy). Sleep is assessed with standard PSG techniques using the 10–20 system [17]. The following variables are continuously measured and recorded throughout the night: Fz, Cz, F4-M1, C4-M1, O2-M1, F3-M2, C3-M2 and O1-M2, left and right electrooculogram, chin electromyogram, left and right anterior tibialis electromyogram and electrocardiogram.

Hypopnea, obstructive and central apnea are assessed thanks to respiratory efforts with thoracic, abdominal inductance plethysmography (SleepSense, S.L.P. Inc., Elgin IL 60,124 USA) and with airflow, which is measured by a nasal pressure cannula (ThermoCan, SleepSense, S.L.P Inc., Elgin, IL, USA). Snoring is scored by filtration of the nasal pressure signal. Peripheral oxygen saturation (SpO_2_) and heart rate (HR) are recorded by pulse oximetry (Nonin medical, Inc. Plymouth, MN, USA). A sensor is placed on the chest and enables detection of the body position during recording.

Patients are instructed to maintain their habitual sleep patterns and asked to record the time of lights-out and the time immediately upon waking the next morning. PSG data are recorded directly to a data acquisition, storage, and analysis system (SleepRT™ software Suite Version 4.04.02 Buil5185; OSG, Belgium). The electroencephalogram (EEG) recordings are visually scored in 30-second periods, to obtain the overnight pattern of sleep stages, and respiratory events in 3-min periods for airflow by an experienced board-certified sleep physician using the American Academy of Sleep Medicine’s standard rules.

The following sleep parameters are recorded: bedtime (h:min), wake-up time (h:min), time in bed (TIB, h), total sleep time (TST, h), sleep efficiency (SE, %), sleep onset latency (SOL, min, time from lights out to sleep onset, defined as the 1st epoch of any sleep stage (min), wake after sleep onset (WASO, min, number of minutes scored as wake-up time during sleep period, min), number of awakenings (nb), awake time (%), total time in stage 1 sleep (TTN1, min), percentage of stage 1 sleep in TST (N1, %), total time in stage 2 sleep (TTN2, min), percentage of stage 2 sleep in TST (N2, %), total time in stage 3 sleep (TTN3, min), percentage of stage 3 sleep in TST (N3, %), total time in rapid-eye movement sleep (TTREM, min) and percentage of REM sleep stage in TST (REM, %).

#### Objective sleep parameters

Patients wear an actimeter on their non-dominant wrist (ActiGraph® GT3X-BT, Pensacola, FL, USA) from bedtime until waking up, for seven consecutive nights. A sleep diary is used, as a complement to actigraphy, to determine when patients are awake and when they are lying down attempting to sleep. The sleep diary collects habits and sleep hygiene over an extended period of two weeks. Patients indicate bedtime, wake-up time, awakenings, periods of sleep and sleepiness, and consumption of drugs. In addition, the subjective quality of sleep and awakening are indicated.

Data from actigraphy are downloaded and analyzed on ActiLife software (ActiGraph®, Pensacola, FL, USA). All records are scored as asleep unless the sleep diary indicates that the patient is lying in bed attempting to sleep. Data are downloaded and analyzed using the Cole-Kripke algorithm on ActiLife software in 60 s epochs. Once the bedtime and wake-up time are defined, automatic Cole-Kripke algorithm analyses are run to extract the following sleep parameters: bedtime (h:min), wake-up time (h:min), SOL (the first minute that the algorithm scores “asleep”), TIB, TST, WASO, awakenings (the number of different awakening episodes as scored by the algorithm), average awakening (the average length, in minutes, of all awakening episodes), total counts (the total actigraphy counts summed together for the entire sleep period), sleep efficiency (SE, number of sleep minutes divided by the total number of minutes the subject is in bed x 100: TST/TIB x 100).

#### Insomnia evaluation

Insomnia is diagnosed per patient-reported symptoms in accordance with criteria from the DSM-5 [6]. DSM-5 classifies insomnia as a subjective complaint with difficulty falling asleep and/or nocturnal awakening and/or early waking despite adequate opportunity for sleep. This condition causes distress and impairment in daytime functioning such as fatigue/low energy, daytime sleepiness, impaired attention/concentration, mood disturbances, amongst other impairments. These disturbances with sleep are not related to any other medical disorder, substance use, or prescription use, and the symptoms must be present at least three nights per week for at least three months.

Insomnia is also assessed using the Insomnia Severity Index (ISI). The ISI is a valid diagnostic screening tool for detecting insomnia and correctly identifies people with DSM-5 defined insomnia disorder [18]. It has also been validated in cancer populations [19]. The ISI comprises seven items that assess the severity of sleep-onset and sleep-maintenance difficulties (both nocturnal and early morning awakening), satisfaction with current sleep pattern, interference with daily functioning, noticeability of impairment attributed to the sleep problem and degree of distress or concern caused by the sleep problem [20, 21]. Each item is rated on a five-point Likert scale (0 = not at all; 4 = extremely) and the usual recall of the “last month”. Total scores range from 0 to 28 with high scores indicating the greatest insomnia severity. The total score is interpreted as follows: absence of insomnia (from 0 to 7); sub-threshold insomnia (from 8 to14); moderate insomnia (from 15 to 21) and severe insomnia (from 22 to 28).

#### Sleep quality and disturbances

The PSQI is a widely used self-reported measure of sleep quality in clinical populations. This scale consists of 24 questions to be rated, relating to the past month (0–3 for 20 items while 4 items are open-ended), 19 of which are self-reported and 5 of which require secondary feedback from a room or bed partner [22]. Only 19 items (15 rated 0–3 and 4 open-ended) are used for the evaluation of sleep quality as perceived by the patients. The open-ended items are also scored as categorical values (rated 0–3) as per the range of values reported by the patients. These 19 self-reported questions are then used to generate scores, which range from 0 (no difficulty) to 3 (severe difficulty), representing the PSQI’s seven components: subjective sleep quality, sleep latency, sleep duration, habitual sleep efficiency, sleep disturbances, use of sleep medications, and daytime disturbance. The total score is generated by the summation of the component scores, and can range from 0 to 21, with a higher score corresponding to a reduced sleep quality. A cutoff score of 5 or 8 represents a “poor” sleeper in the general population or in cancer patients, respectively. A change of 3 points or more on the PSQI is considered a minimal clinically important difference. Brackhaus et al. [23] showed the PSQI to have a high test–retest reliability and a good validity for patients with primary insomnia.

#### Sleepiness

The Epworth Sleepiness Scale (ESS) is a simple self-report scale used to assess daytime sleepiness. The scale asks the respondent to rate the probability of falling asleep in a variety of situations such as reading, watching TV, sitting inactive in public place, or while riding in a car, resting in the afternoon, talking to someone, sitting quietly after lunch, stopping in traffic. A 4-point rating scale is used ranging from 0 (no chance of dozing) to 3 (high chance of dozing). The scores are summed to determine a total ESS score, which ranges between 0 and 24. The higher the score, the higher the person’s level of daytime sleepiness. A cut-off value of 10 (ESS total score > 10) is usually considered to detect excessive daytime sleepiness that could be a sign of a sleep disorder or medical problem. The ESS has been widely used in a variety of studies with normal sleepers and sleep disorders patients.

#### Anxiety and depression

Patients complete the Hospital Anxiety and Depression Scale (HADS) [24]. This scale is a 14-item self-report instrument with 7 questions related to anxiety and 7 others to depressive symptoms. The answer format offers four response options, which are scored with values ranging from 0 to 3 (0 = not at all to 3 = nearly all the time). Thus, each subscale (depression and anxiety) yields a score values between 0 and 21. HADS defines three ranges for both scales: 0–7 (no symptoms), 8–10 (doubtful symptomatology), and 11–21 (some symptomatology). It is possible to calculate a HADS total score by summing the anxiety and depression items [25].

#### Circadian typology

Patients complete the Horne and Östberg Morningness-Eveningness Questionnaire (MEQ), which is widely used to determine diurnal preference [26]. The MEQ is a 19-item questionnaire. The total score gives 5 typologies: definite morning type (score 70–86), moderate morning type (score 59–69), intermediate (score 42–58), moderate evening type (score 31–41), definite evening type (score 16–30).

#### Dim light melatonin onset (DLMO)

The time of onset of melatonin secretion in light condition (< 50 lx), in the evening, called the dim light melatonin onset (DLMO) phase is assessed for each patient. Patients wear glasses composed of acrylic lenses and a UV-530 filter (Bio Optik, Lux Therapie, Strasbourg, France), filtering the blue component of light, 1 h before the beginning of the first sampling. Patients are prompted to give 5 to 6 saliva samples of 2 ml every 30 min using Salivettes (Sarstedt, Newton, NC) 4 h before their average bedtime. The participants tip the cotton swab from the Salivette into their mouths and roll the cotton swab in their mouths for up to 5 min until saturated, before spitting it back into the Salivette. Toothpaste or mouthwash are not allowed during the phase assessments. Small snacks and fluids are permitted, except in the 20 min before each sample, and participants are required to rinse and brush their teeth with water while remaining seated 10 min before each sample if they had consumed food or drink. Participants are not permitted to consume any alcohol or caffeine at least 24 h before each phase assessment.

After collection, saliva samples are frozen at -20 °C, then at -80 °C until analysis. Salivary melatonin is determined by a Direct Saliva Melatonin ELISA kit from NovoLytiX GmbH society (Witterswill, Switzerland). Saliva specimens from a given subject are run with the same assay kit [27, 28]. The method used is the relative threshold 2-SD, defined as 2 times the standard deviation of the mean of the 3 baseline values. The relative threshold method has been shown to have low intra-individual variability [29, 30].

#### Core body temperature rhythm

Circadian core body temperature (CBT) rhythm is established as a major influence on the sleep/wake cycle. CBT is measured using four iButtons (Humeau Laboratories, France) placed at each wrist and infraclavicular fossa, secured with a Comfell protective film. Recording begins in the middle of the afternoon and continues for 48 h with an acquisition frequency of one measurement every 10 min. Data collection is obtained from the Thermotrack software (ref: 303.000105.47) [31].

The analysis of the circadian rhythm of CBT [acrophase (h:min), batyphase (h:min), period (h) and mesor (°C), amplitude (°C)] are carried out by a Cosinor technique (Time Series Analysis Seriel Cosinor, Version 6.3, Soft Technology Expert, Laboratory of Applied Statistics and BioMedical Computing, Richelieu, France). Acrophase and batypase are the measures of the time of overall high and low values recurring in each cycle. Period represents the duration of one cycle, MESOR (Midline Estimating Statistic Of Rhythm), is a rhythm-adjusted mean, and amplitude is a measure of half the extent of predictable variation within a cycle.

#### Fatigue

Fatigue is assessed by the Multidimensional Fatigue Inventory 20 (MFI-20), which is a 20-item self-assessment questionnaire measuring subjective fatigue perceived by patients according to the following sub-scales: general fatigue, physical fatigue, mental fatigue, reduced motivation and reduced activity [32, 33]. Each subscale uses a 5-point Likert scale ranging from 1 (strongly agree) to 5 (strongly disagree). A higher total score indicates a higher level of fatigue.

#### Pain

The Brief Pain Inventory short form (BPI-SF) is a self-administered questionnaire used to evaluate pain severity and pain interference [34]. The pain severity factor consists of four items that require patients to measure the severity of their current pain on a scale of 0 to 10 in order to capture the worst, average, and least pain intensity they felt in the previous 24 h. A value of “0” indicates “no pain” while “10” indicates “pain as bad (excruciating) as patients can imagine”. Pain interference involves 9 items on general activity, mood, walking ability, normal work, relationships with other people, sleep and enjoyment of life, rated on a numerical scale from 0 = “Does not interfere” to 10 = “Interferes completely”. The scale comprises a diagram of a human figure for locating areas of pain, questions about pain medications and the percentages of pain relief achieved with medications in the last 24 h.

#### Biological variables

Blood samples are drawn by venous puncture for analysis inflammatory variables.

Then, plasma is separated by centrifugation (at 4 °C, at a speed of 3500 rpm for 15 min), and transferred into plastic tubes of 2 mL. These tubes are frozen at -20 °C for a maximum of 3 months, then kept at -80 °C until analysis. C-reactive protein (CRP), interleukin 6 (IL-6), tumor necrosis factor alpha (TNF-α) are measured by immune nephelometry method.

#### Cardiorespiratory fitness

Patients perform a maximal graded exercise test (GET) on a cycle ergometer (Ergoselect 200; Ergoline; Bitz, Germany), under the supervision of a cardiologist. After a warm-up period of 3 min at 30 watts, intensity gradually increases by 10 watts every minute until exhaustion (V˙O_2_ peak). Pedaling frequency is maintained around 50 rpm. The exercise is interrupted when two of the three maximum criteria are reached: a decrease of cadence below 50 rpm, a respiratory exchange ratio value exceeding 1.0, attainment of 100% of age-predicted maximal HR (220-age). As soon as the maximum effort is reached, an active recovery period of 10 min without intensity is carried out.

Before, during exercise and recovery, HR is recorded using multichannel electrocardiograms (ECGs) (CASE P2, GE Healthcare, Buckinghamshire, UK). Ventilatory parameters are recorded continuously in real time, cycle by cycle with a gas analyzer system (MGC-CPX System; MGC Diagnostics Corporation, Saint Paul, MN, USA), which is calibrated using gases of known concentration. Oxygen uptake (V˙O_2_), carbon dioxide production (V˙CO_2_), respiratory exchange ratio (V˙CO_2_/V˙O_2_), and ventilation per minute (V˙E) are followed and monitored.

The ventilatory threshold 1 (VT_1_) and 2 (VT_2_) are determined, blinded according to the method of Wasserman using respiratory oxygen (V˙E⁄V˙O_2_) and carbon dioxide equivalents (V˙E⁄V˙CO_2_). The VT_1_ corresponds to the first increase in V˙E⁄V˙O_2_ without a concomitant increase in V˙E⁄V˙CO_2_, whereas VT_2_ corresponds to the second increase in V˙E⁄V˙O_2_ and the first increase in V˙E⁄V˙CO_2_ [35]. VT_1_ and VT_2_ are used as intensity of work for training. Perceived exertion is evaluated by the Borg Rating of Perceived Exertion (RPE) scale, from the beginning to the end of the graded test.

Before, at maximal effort and after 5 min recovery, blood gas (SaO_2_, PaO_2_, PaCO_2_, pH and Excess Bases) and lactate levels are collected using a micro-prick on the earlobe (EML105 Radiometer, Radiometer Medical Aps, Brønshøj, Denmark). These samples are analyzed using the RapidPoint® 500 system (Siemens Healthcare Diagnostics, Erlangen, Germany).

Systolic and diastolic blood pressures are monitored at rest and throughout the trial until the end of recovery, using an automatic sphygmomanometer (SunTech Tango+, Sun Tech, Morrisville, NC, USA).

#### Supervised exercise program

**Fig. 3:**
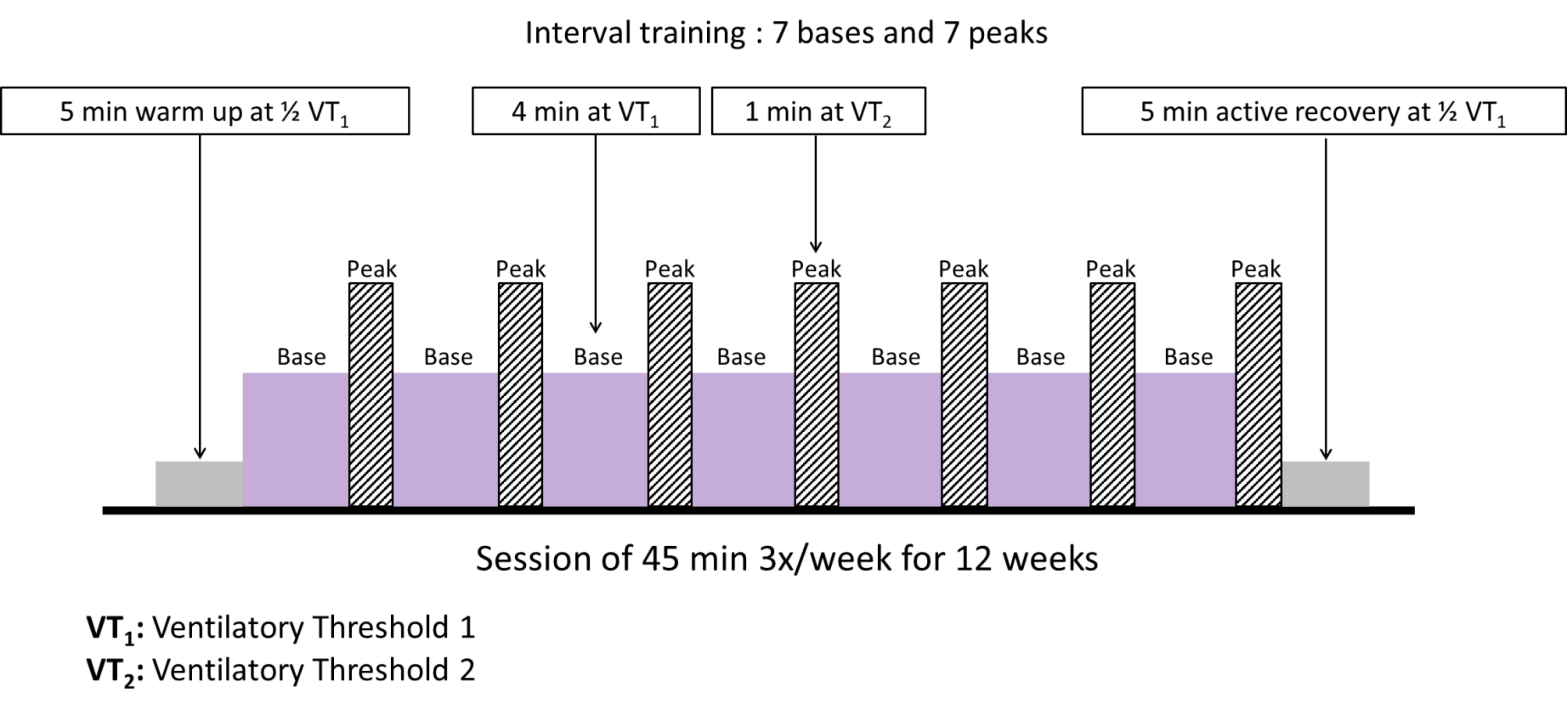
Supervised exercise program

The supervised exercise program is performed on a cycle ergometer, for 3 sessions of 45 min per week for 12 weeks. It consists of an interval training exercise that starts with a 5-minute warm-up (intensity equal to ½ VT_1_), followed by 7 successive training session alternating 4 min of moderate work (intensity equal to VT_1_) and 1 min of intense work (intensity equal to VT_2_). An active recovery is performed for 5 min (at ½ VT1) (Fig. 3). Workloads are alternately readjusted by 10 watts to maintain target HR over time. HR is monitored by a pulse oximeter and fatigue by a visual analogue scale.

#### Follow-up of physical activity

From T0 to T6, physical activity level is followed using a Steel HR watch (Withings, Issy-les-Moulineaux, France), connected to the “Withings Health Mate” application on a mobile phone. The instructor downloads the “Withing Health Mate” mobile application and synchronizes it with the watch. He/she then gives information to the patient on how to use the watch and the mobile application. The application needs to be regularly used by the patients to transfer activity data via Bluetooth. All patients have to wear the watch from the time they get up until they go to bed. They have to schedule each session of physical activity practiced (e.g., walking, muscle strengthening, running, swimming, cycling), during which the distance, duration, intensity, maximum and average HR are collected. The watch also records the number of steps and calories burned, and the daily distance. All data are exported via the application.

## Statistical analyses

### Sample size

To detect a difference of TST of 10% compared to baseline value (considering a TST of 420 min), between the two groups, at T3, a Z-test with a type I error of 5% and a statistical power of 80% will be used. A total of 57 patients including 29 in CG and 28 in TG are required.

Two interim analyses for the efficacy and futility using the Lan-Demets method (O’Brien Fleming boundaries) to estimate the alpha spending function will be performed at 33% and 66% of the information fraction (after enrolment of 19 and 38 evaluable patients). With 10% of non-evaluable patients, 64 patients should be randomized. The primary objective will be done in modified intention-to-treat (miTT): all randomized and evaluable patients for the TST at T0 and T3.

### Data analysis

Statistical analyses will be performed with SAS v9.4, and R v4.1. All primary statistical analyses will be conducted on the miTT population. A p-value < 0.05 will be considered statistically significant for the primary and secondary endpoints.

Patient characteristics will be described for all demographic and clinical data recorded at inclusion in the overall population, miTT population and according to group. Qualitative variables will be described with absolute numbers and percentages. Continuous variables will be reported using mean, standard deviation (SD), median, quartile 1-quartile 3 (q1-q3), min-max.

The comparison of average total sleep time for primary endpoint will be performed with Z-test. Comparisons for secondary endpoints will be carried out using Student’s t-test, Wilcoxon rank-sum test, analysis of variance (ANOVA), and Kruskall-Wallis test, as appropriate. The association of qualitative variables will be carried out using chi-square or Fisher’s exact tests, as appropriate.

## Discussion

To the best of our knowledge, the present FATSOMCAN study is the first randomized study to assess the effects of an exercise program, combining high and moderate intensities, on insomnia and other side effects in breast cancer patients during chemotherapy and after a follow up of 3 months post-exercise intervention. Although it has been shown that exercise leads to several positive outcomes in oncology, it remains unknown to date whether exercise is a valuable option for improving sleep, which is frequently disturbed in cancer patients.

The severity and frequency of insomnia in the breast cancer population deserve special attention in terms of diagnosis, and effective and appropriate management. The importance of treatment stems from the knowledge that insomnia results in more severe fatigue, leads to mood disturbances, contributes to immunosuppression, alters quality of life, and potentially affects the course of the cancer [36–38]. Insomnia in cancer may be due to several causes, which can persist over time. Accordingly, the 3P behavioral model, also known as the Spielman Model [39], delineates how insomnia occurs acutely, but how acute insomnia becomes chronic and self-perpetuating. Savard and Morin [40] distinguished between three types of factors that can influence insomnia: enduring factors that predispose an individual for sleep problems, acute factors that precipitate the onset of sleep problems, and factors that perpetuate the maintenance of sleep problems. Savard et al. [41] further showed that the prevalence rate of the combined insomnia syndrome and symptoms was 69.6% at the time of diagnosis and 59.6% at 2 months after, with higher values for breast cancer. Sleep disturbances need to be thought of as part of the symptom cluster often associated with breast cancer. The concept of symptom clusters, including poor sleep/insomnia, fatigue, psychological distress, depression, and pain, has been described in several studies. The more symptoms within the symptom cluster the patients experience before the start of chemotherapy, the worse the symptoms they experience during chemotherapy [42–44]. Gherman et al. [45], who examined insomnia in the context of breast cancer, reported that approximately 50% of insomnia severity was related to the symptom cluster, with the rest being unique to insomnia. Nevertheless, it has been repeatedly reported in literature that insomnia is strongly correlated with fatigue in breast cancer survivors [46–48]. Schultz et al. [49] suggested that focusing on sleeping problems might be beneficial for fatigue symptoms in those women. The current study might reveal more insights into the importance of a good sleep on multiple symptoms in breast cancer patients.

Among available treatment options for insomnia, hypnotic medications are the most commonly used. Nevertheless, some drawbacks of these medications include their adverse side effects, risk of tolerance, dependence and insomnia rebound. In addition, in the context of breast cancer, there is potential reluctance by patients to take additional medications.

Furthermore, there is a growing body of evidence about the effectiveness of CBT for insomnia (CBT-I). Its efficacy has been demonstrated in the specific context of cancer [50, 51]. Its benefits are comparable to, or better than those observed with pharmacotherapy, in patients with insomnia [52, 53].

Over the past few years, research evidence has highlighted the benefits of physical exercise in reducing side effects in patients undergoing chemotherapy and have reported an improvement in physical functioning, cardiac function, body composition, bone density, cognitive function, fatigue, psychological disorders, immune function and quality of life [54–56]. Conversely, to the best of our knowledge, very few studies have investigated the effects of an exercise program on insomnia in breast cancer. Kreutz et al. [57] showed that physical activity (i.e., walking, aerobic exercise, resistance exercise or a combination of both) tended to outperform mind-body exercise (i.e., yoga, Tai Chi and Qigong) during treatment, with significant effects on subjective sleep outcomes. Mercier et al. [13] reported, in their meta-analysis, the need to assess the effect of physical exercise on sleep disturbances to confirm the available empirical evidence and to determine to what extent exercise is beneficial as a sleep enhancing intervention in cancer patients. Indeed, due to the heterogeneity of studies (varying small sizes, samples of mixed cancer diagnoses, different types or doses of exercise, exercise supervised or not), no consensus exists regarding the optimal exercise prescription against insomnia.

The FATSOMCAN study has several strengths including: (1) a multicenter randomized controlled design using clinical level of insomnia at baseline as an inclusion criterion, (2) a large sample size, (3) evaluation of sleep as the main outcome, (4) objective sleep assessments using ambulatory polysomnography and actimeter, (5) follow-up variables to assess the sustainability of therapeutic gains over time.

Furthermore, we propose a supervised endurance training program that alternates moderate and high intensities, with personalized target intensities, adjusted according to the physical characteristics of the subject. This type of training has already been proposed to patients presenting cardiovascular disease or metabolic syndrome [58–61], with a growing body of evidence emerging in cancer patients [16]. Training has been associated with many benefits, such as improving physical functioning and fitness (i.e., V˙O_2_ peak), reducing side effects of cancer treatments, preventing bone loss and weight gain, increasing muscle strength, decreasing cancer-related fatigue and improving quality of life [16, 62–66].

While exercise is safe and well tolerated by patients undergoing cancer treatments or in the rehabilitation phase [67], findings also show that knowing, and managing insomnia with physical activity offers interesting therapeutic prospects for cancer treatment. However, most studies use only questionnaires without recording objective sleep using polysomnography [57, 68].

This clinical trial will provide additional evidence regarding the effectiveness of physical exercise during chemotherapy in minimizing insomnia, improving sleep quality, reducing fatigue, pain, anxiety, increasing cardio-respiratory fitness and health-related quality of life. If shown to be effective, exercise intervention programs could be welcome addition to the standard program of care offered to patients with breast cancer.

## Data Availability

The datasets used and/or analysed during the current study available from the corresponding author on reasonable request.

## Abbreviations

BPI-SF: Brief pain inventory short form
CBT: Core body temperature
CBT-I: Cognitive behavioral therapy for insomnia
CG: Control group
CRP: C-reactive protein
DSM-5: Diagnostic and statistical manual of mental disorders
ECG: Electrocardiogram
EEG: electroencephalogram
ESS: Epworth sleepiness Scale
GET: graded exercise test
HADS: Hospital Anxiety Depression Scale
HR: Heart rate, IL-6:interleukin 6
TNF-alpha: tumor necrosis factor alpha
ISI: Insomnia severity index
IT: Interval training
ITT: Intention-to-treat
MEQ: Morningness-eveningness Questionnaire
MFI-20: Multidimensional fatigue inventory 20
mITT: Modified intention-to-treat
MTB: Multidisciplinary tumor board
PaCO_2_: Partial pressure of carbon dioxide in arterial blood
PaO_2_: Partial pressure of oxygen in arterial blood
V˙O_2_ Peak: Peak oxygen uptake
pH: Hydrogen potential
PSG: Polysomnography
PSQI: Pittsburgh Sleep Quality Index
REM: Rapid eye movement
RPE: Rating of Perceived Exertion
SaO_2_: Arterial oxygen saturation
SD: Standard deviation
SE: Sleep efficiency
SOL: Sleep onset latency
SpO_2_: Peripheral capillary oxygen saturation
TG: Training group
TIB: Time in bed
TST: Total sleep time
TTN1: Total time in stage 1 sleep
TTN2: total time in stage 2 sleep
TTN3: total time in stage 3 sleep
TTREM: Total time in rapid-eye movement sleep
V˙CO2: Carbon dioxide flow
V˙CO2/V˙O2: Respiratory exchange ratio
V˙E: Ventilation minute
V˙E⁄V˙CO2: Respiratory equivalents in carbon dioxide
V˙E⁄V˙O2: Respiratory equivalent in oxygen
V˙O2: Oxygen consumption
VT1 and VT2: Ventilatory thresholds 1 and 2
WASO: Wake after sleep onset
